# Intramuscular Diphenhydramine Does Not Affect Acute Doxorubicin Infusion-Related Arrhythmia Number or Severity in a Prospective Crossover Study in Canine Lymphoma: A Pilot Study

**DOI:** 10.3389/fvets.2020.00368

**Published:** 2020-07-17

**Authors:** Jennifer Lindley Willcox, Catherine Belanger, Jenna Hart Burton, Lydia Yu, Yu Ueda, Lance C. Visser, Katherine Skorupski, Joshua A. Stern

**Affiliations:** ^1^Department of Surgical and Radiological Sciences, School of Veterinary Medicine, University of California, Davis, Davis, CA, United States; ^2^Department of Medicine and Epidemiology, School of Veterinary Medicine, University of California, Davis, Davis, CA, United States; ^3^Department of Clinical Sciences, College of Veterinary Medicine, North Carolina State University, Raleigh, NC, United States

**Keywords:** histamine, cardiac, chemotherapy, Adriamycin, Benadryl

## Abstract

**Background:** Doxorubicin (DOX) is one of the most effective chemotherapeutics for canine high-grade lymphoma. In addition to dose-dependent chronic cardiotoxicity, DOX can trigger acute cardiac arrhythmias during drug infusion. Diphenhydramine premedication is commonly used, as histamine release is a proposed mechanism for DOX-associated arrhythmogenesis.

**Hypothesis/Objectives:** The study objectives were to evaluate the incidence and severity of DOX infusion-related cardiac arrhythmias in dogs with high-grade lymphoma and evaluate the effect of diphenhydramine premedication on arrhythmia frequency and severity during and after DOX infusion.

**Animals:** Twenty-two client-owned dogs with cytologically/histopathologically confirmed high-grade lymphoma were recruited, of which 19 were enrolled and 9 completed the study.

**Methods:** Dogs were screened by echocardiogram and concurrent electrocardiogram for this randomized prospective crossover study. Group A received no premedication for DOX #1 and was premedicated with diphenhydramine for DOX #2; Group B received diphenhydramine with DOX #1 and no premedication for DOX #2. For both visits, Holter monitor data were collected for 1 h pre-DOX and 3 h post-DOX administration.

**Results:** Nineteen dogs were enrolled and 9 dogs [Group A (5), Group B (4)] completed the protocol. There was no statistical difference between the DOX alone and DOX + diphenhydramine when evaluating the total number of ventricular premature complexes (VPCs, *P* = 0.34), change in VPCs/hour (*P* = 0.25), total number of atrial premature complexes (APCs, *P* = 0.5), change in APCs/hour (*P* = 0.06), or ventricular arrhythmia severity score (*P* > 0.99).

**Conclusions and clinical importance:** This study demonstrates that in these dogs with rigorous pretreatment cardiovascular screening, DOX infusion did not induce significant arrhythmias. Furthermore, these data suggest that, with this screening approach, diphenhydramine may not alter the arrhythmia number or severity in canine DOX recipients.

## Introduction

Doxorubicin (DOX) is one of the most widely used antineoplastic drugs in veterinary and human medicine and demonstrates efficacy against a variety of cancers such as lymphoma and solid tumors like hemangiosarcoma (1–6). Unfortunately, like most chemotherapeutics, DOX can also be associated with adverse effects on tissues and organs. Of these, DOX dose-dependent cardiotoxicity is well-known and can greatly impede its clinical use (7–15).

In humans and dogs, there is significant individual variation in susceptibility regarding DOX cardiotoxicosis, and in general, dogs are considered to be more sensitive than humans (8, 16–19). Many studies have investigated DOX-associated cardiotoxicity, and they are commonly divided into chronic and acute categories. The most important chronic effect is a dose-dependent and irreversible cardiomyopathy (20, 21). This can lead to congestive heart failure and ventricular arrhythmias, and is commonly associated with a poor prognosis, especially in canine patients (22–26). Although cardiotoxicity can occur with any cumulative dose, it is most commonly seen with doses that exceed 180 mg/m^2^ (or >6 doses in most dogs) or in humans ≥450 mg/m^2^ (7, 9, 11, 12, 18, 19, 27–29). Acute effects mainly include arrhythmias that are transient and rarely life-threatening, although left ventricular failure, pericarditis, myocarditis, and sudden cardiac death have been reported in people (9, 11, 19, 26, 27, 30–36). In general, acute cardiotoxicities are associated with the peak level of DOX and/or rapid infusion (28, 33, 37). These effects vary from non-specific electrocardiogram ST changes to atrial and ventricular arrhythmias (38–41). In humans, the incidence of acute cardiotoxicity causing arrhythmias ranges from 11 to 40% (2, 19, 33, 38, 42–46).

The underlying cause of acute DOX-induced cardiotoxicity remains unclear. Numerous mechanisms have been proposed in which some reports suggest that histamine and catecholamine release could play a role in the acute cardiotoxic effects (28, 47). Hypotension and allergic reactions related to the drug infusion rate may also play a role in arrhythmogenesis (12, 48, 49).

Considering the previously documented systemic release of histamine during DOX infusion and its potential role in arrhythmogenesis, the use of antihistamine premedication prior to DOX infusion is common practice (28, 41, 50, 51). Currently, there is a paucity of evidence to support or refute the use of antihistamines for infusion-related cardiac arrhythmias in clinical canine patients. The study objectives were to evaluate the incidence and severity of DOX infusion-related cardiac arrhythmias in dogs with high-grade lymphoma, and evaluate the effect of the antihistamine, diphenhydramine, premedication on the arrhythmia number and severity during and after DOX infusion. We hypothesized that diphenhydramine would not impact the number or severity of cardiac arrhythmias observed during DOX infusion in canine lymphoma patients.

## Materials and Methods

This was a prospective, randomized, crossover design study that enrolled client-owned dogs diagnosed with high-grade lymphoma presented to the University of California, Davis Veterinary Medical Teaching Hospital (UCD-VMTH) in accordance with the UCD Institutional Animal Care and Use Committee (protocol #19015) from 10/2015 to 10/2018. Dogs were included if they had a cytologic or histopathologic diagnosis of high-grade lymphoma, were receiving a DOX-based multiagent chemotherapy protocol, which at this institution during that timeframe was either a 6-month or 19-week CHOP [cyclophosphamide (cyclophosphamide, Hikma, Columbus, OH), doxorubicin (doxorubicin hydrochloride; Pfizer, New York, NY), vincristine (Hospira, Lake Forest, IL), and prednisone (prednisone, PAR Pharmaceuticals, Chestnut Ridge, NY)] protocol, had not been previously treated with DOX, had a performance status of 0 or 1 (modified Eastern Cooperative Oncology Group), and weighed > 15 kg (4, 52). This body weight restriction was based on this institution's protocol to administer DOX at 30 mg/m^2^ for dogs > 15 kg and 1 mg/kg for dogs ≤ 15 kg (4, 52–55). Concurrent use of corticosteroids was permissible as per the overseeing clinician's recommendations. Specific supplements (chondroitin sulfate, vitamins, essential fatty acids, and glucosamine) were also permitted if administered for >30 days prior to enrollment, and no new supplements or oral antihistamines were allowed during the study period. At this institution, drugs to manage adverse events such as gastrointestinal toxicity would generally include ondansetron (ondansetron hydrochloride, Rising Health Saddle Brook, NJ), maropitant (Cerenia, Zoetis, Kalamazoo, MI), and/or metronidazole (metronidazole hydrochloride, Blue Point Laboratories, Montgomeryville, PA) or, in the event of clinically important myelosuppression, enrofloxacin (Baytril, Bayer, Shawnee Mission, KS) or amoxicillin clavulanic acid (Clavamox, Zoetis, Kalamazoo, MI) would be prescribed at the discretion of the supervising clinician. Dogs were excluded if they had a clinically significant grade 3 or higher hematologic/biochemical abnormality as defined by the Veterinary Cooperative Oncology Group (VCOG-CTAE 1.1), considered a substage b, received an antihistamine within 72 h of DOX #1, or had significant comorbidities including but not limited to liver or renal insufficiency, heart disease, or any other abnormality deemed incompatible with DOX administration (56). Dogs were also excluded if they were unable to receive DOX at the prescribed 30 mg/m^2^ dose.

Prior to enrollment, all dogs underwent a complete physical examination and cardiologic examination including a complete echocardiogram with concurrent lead 2 electrocardiogram (ECG) for the duration of the echocardiographic study (Philips EPIQ 7C or ie33; Philips Healthcare; Andover, MA). The screening echocardiogram was used to rule out any concurrent systolic dysfunction as judged by the attending cardiologist. Dogs with observed systolic dysfunction or concomitant cardiac disease beyond mild valvular degeneration/insufficiency as reported by the attending cardiologist were excluded from the study. Additionally, dogs with ventricular or supraventricular arrhythmias observed during the concurrent ECG were excluded from the study. Additional diagnostic tests were performed at the discretion of the owner and overseeing oncology clinician (complete blood count, chemistry, and ± urine analysis, thoracic radiographs, and abdominal ultrasound). Clinical information collected for each dog included age, body weight, sex, breed, and staging information.

Once dogs were enrolled, they were randomized into either Group A or Group B according to a predetermined randomization table. Group A dogs received no premedication for DOX dose #1 and were premedicated with diphenhydramine (diphenhydramine HCl; West-Ward, Eatontown, NJ) for DOX dose #2; Group B dogs received diphenhydramine with DOX dose #1 and no premedication for DOX dose #2. For both Groups A and B, the diphenhydramine premedication was administered intramuscularly 30 min prior to the DOX infusion at a dose of 2 mg/kg. A 5-electrode Holter monitor (Burdick Holter monitor; Ventura, CA) was placed as previously described for a total of 4 h in the clinic, which recorded 1 h prior to DOX infusion to serve as a baseline and for 3 h during/post DOX infusion (57). All dogs received their DOX infusion over 20 min via an intravenous catheter placed in a peripheral vein as per UCD-VMTH's protocol. After DOX administration and Holter monitoring, dogs were discharged with standard recommendations and monitoring. On the second visit, all dogs underwent a physical examination and diagnostic tests as per the clinician's discretion. The Holter monitor data for all patients were evaluated by a cardiology resident in training (CB) and confirmed by a board-certified veterinary cardiologist (JS) using commercially available software (Burdick Holter Analysis Software; Ventura, CA). For each Holter dataset, both observers (CB, JS) were blinded to patient information, randomization assignment, and whether or not DOX was given alone or with diphenhydramine premedication. Ventricular arrhythmias were graded using a standardized scoring system derived for this project where 0 represented no ectopy, 1 for only single ventricular complexes, 2 for ventricular couplets or triplets, 3 for polymorphic ventricular ectopic complexes, and finally 4 if paroxysmal or sustained ventricular tachycardia was observed. If clinically relevant arrhythmias were noted by the cardiologist reading the Holter monitor, then therapeutic recommendations were provided.

### Statistical Analysis

Continuous data were both visually inspected and tested for normality using a D'Agostino and Pearson normality test. Parametric data are presented as mean ± SD, and non-parametric data are presented as median and minimum to maximum. Paired data (each patient compared to itself when treated with DOX alone or DOX + diphenhydramine premedication) were tested for differences compared using a Wilcoxon matched-pairs signed rank test for non-parametric data and a paired *t*-test for parametric data. Statistically significant differences were reported at *P* < 0.05. Comparisons were performed for total atrial premature complexes (APCs) and ventricular premature complexes (VPCs) in 4 h, change in APCs and VPCs comparing the number of arrhythmias in hour 1 to the subsequent 3 h, and lastly the ventricular arrhythmia severity score. All statistics were performed using commercially available software (GraphPad Prism Software, versions 7 and 8; La Jolla, CA).

## Results

### Clinical Demographics

**Figure 1:**
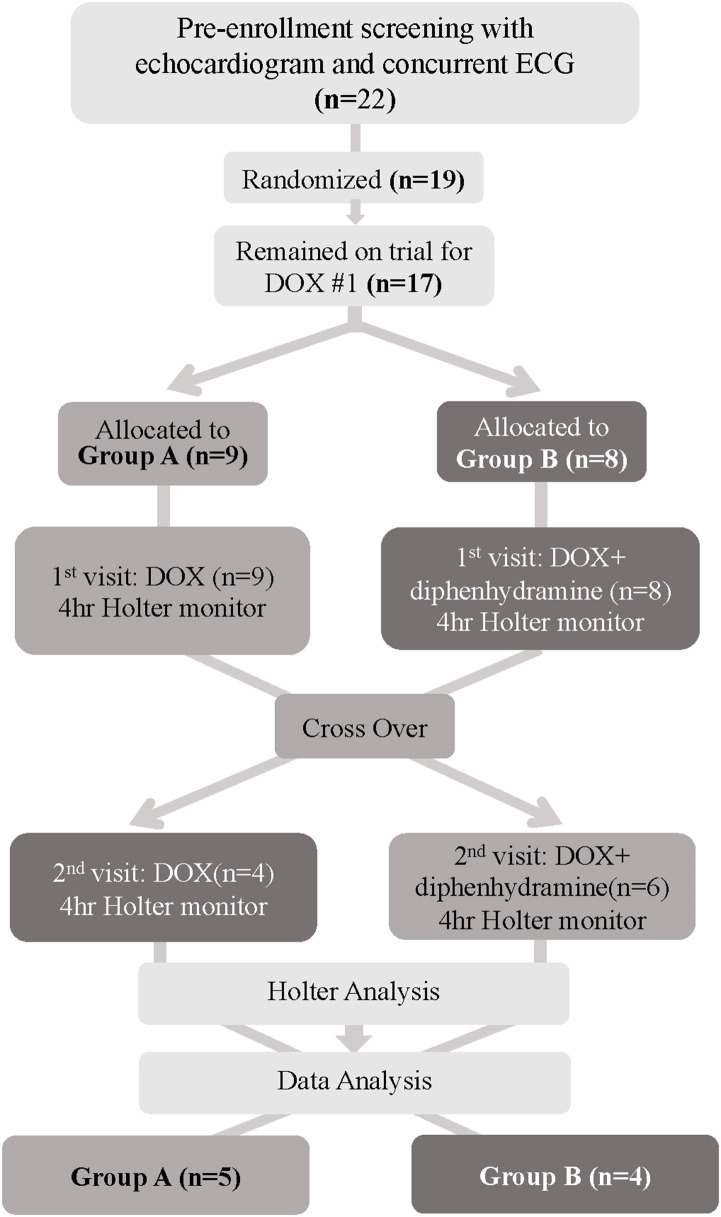
Flowchart depicting patient randomization and progress for dogs enrolled in this trial. DOX, Doxorubicin; ECG, electrocardiogram.

A total of 22 dogs were screened for the trial, and 19 dogs were randomized (Figure 1). Three dogs failed screening with the identification of a significant ventricular arrhythmia in one dog, a heart base mass along with VPCs in one dog, and VPCs due to arrhythmogenic right ventricular cardiomyopathy in one dog. Of the remaining 19 dogs, they had a median age of 7 years (2–13) with 6 spayed females, 10 castrated males, and 3 intact males. Dogs had a median body weight of 31.7 kg (21.6–52.9 kg). Multiple breeds were represented with Labrador retrievers being the most common (6) followed by mixed breed dogs (4), golden retrievers (2), pitbull terriers (2), and one each of Australian shepherd, German shepherd, border collie, Doberman pinscher, and poodle. Based on the World Health Organization criteria for lymphoma, most dogs were considered at least a stage III (9), with three stage IV, five stage V, and two dogs with mediastinal lymphoma; however, not all dogs were fully staged as this was at the discretion of the pet owner and the clinician (58). All dogs received concurrent corticosteroids as part of their chemotherapy protocol for DOX #1 and two dogs remained on corticosteroids at the time of DOX #2.

### Patient Randomization and Attrition (Figure 1)

Of the 19 dogs enrolled and randomized, 2 dogs were removed from the study prior to DOX #1 due to owner withdrawal and lack of pursuit of chemotherapy, leaving 17 dogs. Of the 17 dogs, 9 were randomized to Group A and 8 dogs were allocated to Group B. Group A had 6 dogs remaining at DOX #2, as 3 dogs did not receive the second DOX due to either progressive disease on their chemotherapy protocol (2) or patient non-compliance (1). Group B also experienced attrition of 4 dogs that were removed from the study due to dose reductions in DOX #2 secondary to adverse effects following DOX #1. The adverse events were as follows: the first dog had a grade IV neutropenia and grade 2 vomiting 10 days after DOX #1, the second dog was hospitalized for grade III vomiting and diarrhea 5 days post-DOX #1 and a grade III neutropenia and grade II thrombocytopenia 7 days post-administration, the third dog had grade III diarrhea for which outpatient supportive care was provided 5 days post-DOX #1, and the fourth dog had grade II anorexia, vomiting, and lethargy 2 days post-DOX #1. Finally, once Holter data were evaluated, 1 dog from Group A was identified to have significant ventricular ectopy for their 1-h baseline period and was withdrawn from the study due to recommended antiarrhythmic therapy, leaving a total of 9 evaluable dogs at study completion (Supplementary Table 1). The underlying cause of this dog's preexisting arrhythmia was unknown and could have been secondary to their lymphoma or another cause. Systemic hypersensitivity reactions were not seen in any of the enrolled dogs and thus did not contribute to attrition (Figure 1).

### Holter Data

**Figure 2:**
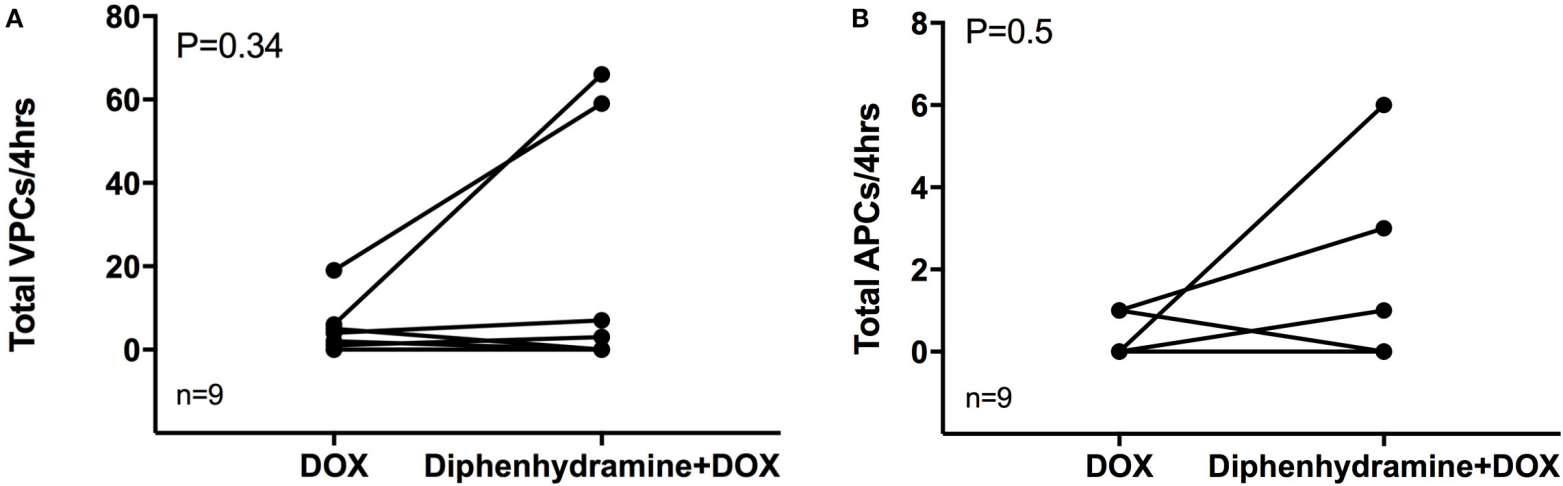
The total number of ventricular premature complexes (VPCs) and atrial premature complexes (APCs) over the observed 4 h is shown in **(A)** and **(B)**, respectively, for each of the nine evaluable dogs in each treatment group [doxorubicin (DOX) alone and DOX + diphenhydramine]. Each animal is represented by a single point in each treatment group and connected with a single line to represent the paired comparisons employed.

**Figure 3:**
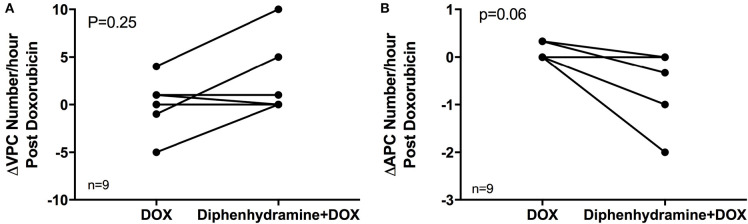
The change in the number of ventricular premature complexes (VPCs) and atrial premature complexes (APCs) comparing the 1 h of pretreatment Holter monitor data to the 3 h during and following doxorubicin (DOX) administration is shown in **(A)** and **(B)**, respectively, for each of the nine evaluable dogs in each treatment group [doxorubicin (DOX) alone and DOX + diphenhydramine]. Each animal is represented by a single point in each treatment group and connected with a single line to represent the paired comparisons employed.

**Figure 4:**
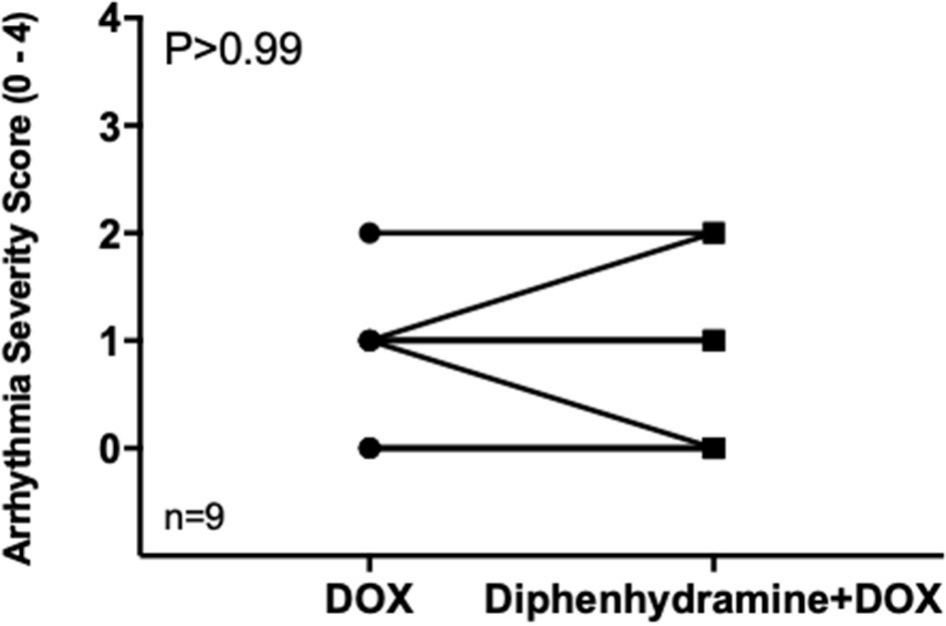
The arrhythmia severity score for ventricular ectopy is shown for each of the nine evaluable dogs in each treatment group [doxorubicin (DOX) alone and DOX + diphenhydramine]. Each animal is represented by a single point in each treatment group and connected with a single line to represent the paired comparisons employed.

Holter data were assessed for frequency of both VPCs and APCs as well as complexity of VPCs using the provided grading scheme. Overall, arrhythmogenesis was low with no significant difference between the total number of VPCs or APCs over 4 h in the DOX alone or DOX + diphenhydramine groups (Figure 2). Dogs had a median of 2 (0 to 19) VPCs for the DOX alone population and a median of 0 (0 to 66) VPCs for the DOX + diphenhydramine dogs (*P* = 0.34). A median of 0 (0 to 1) APC was observed in the DOX alone group and a median of 0 (0 to 6) was observed for the DOX + diphenhydramine group (*P* = 0.5). When the change in the frequency of arrhythmias was assessed comparing the initial baseline hour of Holter monitor data to the subsequent 3 h, there was no significant difference in the change in the number of VPCs [DOX alone median 0, (−5 to 4); DOX + diphenhydramine group median 0, (0 to 10); *P* = 0.25)] or APCs [DOX alone median 0, (0 to 0.33); DOX + diphenhydramine group median 0, (−2 to 0), (*P* = 0.06)] (Figure 3). Arrhythmia severity score for ventricular ectopy was not different between groups [(DOX alone median 1, (0 to 2); DOX + diphenhydramine group median 1, (0 to 2); *P* = 0.99] (Figure 4).

## Discussion

To the authors' knowledge, this is the first clinical trial investigating the effects of the antihistamine, diphenhydramine, on DOX infusion-related cardiac arrhythmias in client-owned dogs. Our study demonstrates that with the pretreatment cardiovascular screening by echocardiogram and concurrent ECG used in this study, DOX infusion did not commonly induce clinically relevant cardiac arrhythmias in this population of dogs. Overall, arrhythmogenesis was low, and there was no significant difference in the total number of VPCs or APCs, change in VPCs or APCs, or the arrhythmia severity score when dogs received DOX alone or DOX + diphenhydramine premedication. Of the 22 screened dogs, 4 (18%) were excluded from the trial for concomitant cardiac disease, and the study completion rate was low with only 9 of 17 (52%) dogs having evaluable data at study completion.

The lack of diphenhydramine impact could be due in part to the current UCD-VMTH clinical practice for DOX administration as a 20-min infusion. Increased adverse events have been reported when DOX is administered as a bolus rather than as an infusion in dogs and people (28, 59). Bristow et al. (28) demonstrated more significant histamine release when DOX was administered as a bolus rather than a slow infusion, but did not evaluate these dogs for concurrent arrhythmogenesis (28). Additionally, histamine increases have been associated with reduced cardiac systolic function; thus, it may remain reasonable to consider premedication with an antihistamine when DOX is used in patients with known cardiac disease (50). The UCD-VMTH protocol also incorporates the use of prednisone as part of the multiagent chemotherapy protocol for lymphoma. Although corticosteroids are known to reduce blood histamine concentration, it was not eliminated from the study as it remains part of standard therapy for lymphoma at this institution and mirrors clinical practice (4, 52, 60). Comparisons were not made between dogs receiving prednisone vs. those that were not due to small sample size.

This prospective study has several limitations to consider. The first of which is secondary to the small numbers and a low completion rate, which could make results subject to a Type II error. The authors acknowledge the diminished completion rate with only 9 dogs (47%) being evaluable of the 19 dogs randomized. Multiple factors contributed to this attrition including adverse events that triggered dose reductions and thus elimination from the trial. The authors deemed maintenance of dose intensity to be necessary to standardize as reductions may decrease the likelihood of side effects in some patients (61). Other dogs went on to progress on their chemotherapy protocol as DOX was no longer efficacious for their disease, and thus, they were eliminated from the trial. Administration of antihistamines outside of the study protocol was prohibited in this study; however, supportive medications to treat chemotherapy-induced adverse effects were permitted. There is potential for these concomitant medications to impact our data if an idiosyncratic reaction were to occur; but it was deemed unethical to withhold supportive care from these client-owned dogs, and none of the drugs routinely utilized have known arrhythmogenic instigation or suppressive properties. Although no dogs experienced a systemic hypersensitivity reaction, another potential limitation would include that this study was not designed to evaluate non-cardiac implications of diphenhydramine omission such as hypotension, facial swelling, wheals, etc. as the incidence rates are generally low when DOX is administered as a slow infusion rather than a bolus (61)]. Future studies would be necessary to identify potential differences with regard to these reactions between diphenhydramine treated and untreated groups. Finally, it is important to acknowledge that our results may not be applicable to a population with less rigorous screening criteria. Regardless of breed and physical examination findings, all dogs in this study had an echocardiogram with concurrent ECG, which may not mimic clinical practice at all institutions.

## Conclusion

These data support that diphenhydramine premedication provided no appreciable benefit in the arrhythmia number or severity in this population of dogs with cardiovascular prescreening.

## Data Availability Statement

All datasets generated for this study are included in the article/Supplementary Material.

## Ethics Statement

The animal study was reviewed and approved by University of California, Davis Institutional Animal Care and Use Committee (IACUC, protocol #19015) from 10/2015 to 10/2018. Written informed consent was obtained from the owners for the participation of their animals in this study.

## Author Contributions

JW, JS, CB, and JB were involved in study idea, design, implementation, data acquisition, data analysis, and manuscript preparation. LY, YU, LV, and KS were involved in study implementation, data acquisition, data analysis, and manuscript preparation. All authors provided approval and accountability for this work.

## Conflict of Interest

The authors declare that the research was conducted in the absence of any commercial or financial relationships that could be construed as a potential conflict of interest.

## Abbreviations

APC: Atrial Premature Complex
DOX: Doxorubicin
UCD-VMTH, University of California, Davis: Veterinary Medical Teaching Hospital
VPC: Ventricular Premature Complex
VCOG-CTAE 1. 1, Veterinary Comparative Oncology Group: Common Terminology of Adverse Events version 1.1.
