# Supplementary material for: Intramuscular Diphenhydramine Does Not Affect Acute Doxorubicin Infusion-Related Arrhythmia Number or Severity in a Prospective Crossover Study in Canine Lymphoma: A Pilot Study
Source: Front Vet Sci. 2020 Jul 17;7:368. doi: 10.3389/fvets.2020.00368 (PMC7379900; doi:10.3389/fvets.2020.00368)
Supplement: Supplementary file 1 [file Table_1.pdf]

Supplementary Table 1

Demographics, group assignment, and dog specific Holter data provided for the 9 evaluable dogs reaching study completion. The total number of ventricular premature complexes (VPCs) and atrial premature complexes (APCs) as well as the arrhythmia severity score for ventricular ectopy for both treatment groups (doxorubicin (DOX) alone and with diphenhydramine premedication) are given for the observed 4 hour time interval.

Dog	Group	Age (yr)	Weight (kg)	Sex	Breed	VPCs DOX alone	VPCs DOX + diphenhydramine	APCs DOX alone	APCs DOX + diphenhydramine	Arrhythmia severity score DOX alone	Arrhythmia severity score DOX + diphenhydramine
1	A	7	41.9	FS	Golden retriever	0	0	0	0	0	0
2	A	5	30.8	MC	Pitbull	6	66	0	6	2	2
3	A	3	30.2	FS	Doberman pinscher	0	0	0	0	0	0
4	A	10	33	FS	Labrador retriever	0	0	1	0	0	0
5	A	8	36.2	MI	German shepherd	5	0	1	3	1	0
6	B	13	28.7	MC	Poodle	1	3	0	0	1	1
7	B	6	26	MC	Mixed breed	2	0	0	1	1	2
8	B	10	31.5	MI	Border collie	19	59	1	0	1	1
9	B	13	26.6	FS	Boxer mix	4	7	0	0	1	1

Abbreviations: Year (yr), kilogram (kg), female spayed (FS), male castrated (MC), male intact (MI)
